# Comprehensive Multi-Omics Analysis Reveals Aberrant Metabolism of Epstein–Barr-Virus-Associated Gastric Carcinoma

**DOI:** 10.3390/cells8101220

**Published:** 2019-10-08

**Authors:** Sang Jun Yoon, Jun Yeob Kim, Nguyen Phuoc Long, Jung Eun Min, Hyung Min Kim, Jae Hee Yoon, Nguyen Hoang Anh, Myung Chan Park, Sung Won Kwon, Suk Kyeong Lee

**Affiliations:** 1 College of Pharmacy, Seoul National University, Seoul 08826, Korea; supercanboy@snu.ac.kr (S.J.Y.); phuoclong@snu.ac.kr (N.P.L.); mje0107@snu.ac.kr (J.E.M.); snuhmkim04@snu.ac.kr (H.M.K.); 2018-23140@snu.ac.kr (N.H.A.); 2 Department of Medical Life Sciences, Department of Biomedicine & Health Sciences, College of Medicine, The Catholic University of Korea, Seoul 06591, Korea; kjygod1@catholic.ac.kr (J.Y.K.); yjh823@catholic.ac.kr (J.H.Y.); pmcreg@catholic.ac.kr (M.C.P.)

**Keywords:** Epstein–Barr-virus-associated gastric cancer, cancer metabolism, transcriptomics, metabolomics, lipidomics

## Abstract

The metabolic landscape of Epstein–Barr-virus-associated gastric cancer (EBVaGC) remains to be elucidated. In this study, we used transcriptomics, metabolomics, and lipidomics to comprehensively investigate aberrant metabolism in EBVaGC. Specifically, we conducted gene expression analyses using microarray-based data from gastric adenocarcinoma epithelial cell lines and tissue samples from patients with clinically advanced gastric carcinoma. We also conducted complementary metabolomics and lipidomics using various mass spectrometry platforms. We found a significant downregulation of genes related to metabolic pathways, especially the metabolism of amino acids, lipids, and carbohydrates. The effect of dysregulated metabolic genes was confirmed in a survival analysis of 3951 gastric cancer patients. We found 57 upregulated metabolites and 31 metabolites that were downregulated in EBVaGC compared with EBV-negative gastric cancer. Sixty-nine lipids, mainly ether-linked phospholipids and triacylglycerols, were downregulated, whereas 45 lipids, mainly phospholipids, were upregulated. In total, 15 metabolisms related to polar metabolites and 15 lipid-associated pathways were involved in alteration of metabolites by EBV in gastric cancer. In this work, we have described the metabolic landscape of EBVaGC at the multi-omics level. These findings could help elucidate the mechanism of EBVaGC oncogenesis.

## 1. Introduction

Gastric cancer (GC) is the fifth most commonly diagnosed form of cancer and the third highest in tumor-associated deaths [1]. The median survival time of gastric cancer patients is only 12 months, and the 5-year survival rate in most areas is just 20% [2]. Approximately 90% of gastric cancers are adenocarcinomas [3], and 5–10% of them are Epstein–Barr-virus (EBV)-associated gastric cancer (EBVaGC) [4,5]. EBV is associated with diverse tumors that originate from mesenchymal cells, lymphocytes, and epithelial cells [6]. Among EBV-associated cancers, EBVaGC is the most common [7]. Among four subtypes of gastric carcinoma, EBVaGC stands out from the others because of its substantial and multifactorial molecular and clinicopathological characteristics. A recent meta-analysis of 33 case–control studies (5290 cases and 4962 controls) concluded that an EBV infection increased the chance of developing GC [8]. However, EBV positivity in GC has been known to be associated with a better clinical prognosis, although the precise mechanisms remain to be elucidated [9,10]. Additionally, the association between EBV and gastric cancer is thought to be a predictive indicator for immunotherapy [5]. In EBVaGC, altered methylation patterns of several tumor suppressor genes, such as *CHD1* and p16*^INK4a^*, are commonly observed and regarded as an essential tumorigenesis mechanism [11]. The alteration in DNA methylation usually leads to the downregulation of tumor suppressor genes, which favors the carcinogenesis of GC [12]. On the molecular level, EBVaGC tends to cluster together and has an extreme CpG island methylator phenotype that makes it distinct from other molecular subtypes [13].

The complementary and integrative analysis of multi-omics platforms has been successfully used to elucidate the mechanisms of several human diseases. For instance, the essential role of *ELOVL2* in the progression of breast cancer was elucidated using a multilayer omics analysis on a spheroid-induced epithelial-mesenchymal transition model [14]. An omics-based translational investigation using publicly available datasets combined with data mining is a novel approach to finding robust biomarkers for the prognosis and diagnosis of pancreatic cancer [15]. Currently, there are arguably three strategies for multi-omics integration and interpretation: post-analysis data integration, integrated data analysis, and systems modeling. Post-analysis data integration and integrated data analysis are versatile strategies for hypothesis generation [16]. Large-scale molecular classification, such as by omics-based signatures, has been suggested for the prognosis and management of GC [17]. Metabolomics has been used to discover metabolic biomarkers for screening, diagnosing, and therapeutically monitoring GC [18]. Also, several pathways that might be associated with gastric cancer, such as the tricarboxylic acid (TCA) cycle, glutaminolysis, and lipid peroxidation, were reported [19]. Similarly, a wide range of metabolites (amino acids, nucleic acids, carbohydrates, and fatty acids) was altered in gastric tumors compared with healthy controls and subjects with benign conditions [20]. However, no clear metabolic signature to distinguish EBVaGC from EBV-negative GC (EBVnGC) has yet been established.

Recently, a comprehensive analysis using data from the Cancer Genome Atlas (TCGA) revealed a significant downregulation of metabolic genes in EBVaGC patients [21]. However, acquiring better insights into the mechanistic process of that downregulation and potential therapeutic targets requires an integrative multi-omics analysis that combines large-scale metabolomics and lipidomics with transcriptomics. In this study, we combined supportive evidence from a transcriptome analysis with complementary metabolome and lipidome analyses to describe the metabolic landscape of EBVaGC. In particular, we confirmed the downregulation of a significant number of metabolic-related genes in EBVaGC. We also conducted polar metabolite profiling (untargeted and large-scale targeted metabolomics) and untargeted lipid profiling using multiple mass spectrometry (MS) platforms. This approach allowed us to describe the metabolic landscape of EBVaGC at the multi-omics level. Furthermore, our work revealed the prognostic effects of those metabolic-related genes. Collectively, our investigation opens a new door for therapeutic development and patient management in clinical practice.

## 2. Materials and Methods

### 2.1. Ethical Consideration

In the present study, we used microarray data from human clinical GC tissue samples (deposited as GSE51575) and cell lines (deposited as GSE135644) in the Gene Expression Omnibus (GEO). GSE51575 contains gene expression intensities from EBV-negative gastric cancer tissue (GCT) and EBV-positive gastric cancer tissue (GCT-EBV), and GSE135644 contains gene expression intensities from EBV-negative AGS (AGS) and EBV-positive AGS (AGS-EBV) cells. The metabolic genes downregulated in a differential gene expression analysis of these two datasets were merged and used in a Kaplan–Meier (KM) survival analysis and a schematization of the metabolic landscape of EBVaGC. This study was approved by the Songeui Medical Campus Institutional Review Board of the Catholic University of Korea (IRB approval number, MC19EEDI0078).

### 2.2. Chemicals and Reagents

LC-MS-grade methanol, acetonitrile, 2-propanol, and water were purchased from Merck (Darmstadt, Germany). LC-MS-grade ammonia, formic acid, ammonium acetate, ammonium formate, methoxyamine hydrochloride, HPLC-grade tert-butyl methyl ether (MTBE), benzoic acid (2,3,4,5,6-*d*_5_), D-fructose (U-^13^C_6_), sodium bicarbonate, trimethylsilylation reagent (N, O-bis-(trimethylsilyl)trifluoroacetamide (BSTFA)/trimethylchlorosilane (TMCS)) (99:1 *v/v*), pyridine, and toluene were purchased from Sigma-Aldrich (St. Louis, MO, USA). Ceramide (Cer) (18:1/17:0), sphingomyelin (SM) 18:1/17:0, lysophosphatidylcholine (LPC) 17:0, lysophosphatidylethanolamine (LPE) 17:1, phosphatidylcholine (PC) (10:0/10:0), phosphatidylethanolamine (PE) (10:0/10:0), triacylglycerol (TG) (17:0/17:1/17:0, *d*_5_), and diacylglycerol (DG) (18:1/2:0) were purchased from Avanti (Alabaster, AL, USA). RPMI-1640 medium, penicillin-streptomycin, amphotericin B, and G418 were acquired from Gibco^®^ (Carlsbad, CA, USA). Fetal bovine serum was purchased from Corning^®^ Cellgro^®^ (Corning, NY). RNAiso Plus was purchased from TaKaRa (Kusatsu, Shiga, Japan). Oligo(dT), *EBNA1*, and *GAPDH* primers were purchased from Macrogen (Seoul, Korea). A SYBR green qPCR kit was purchased from Enzynomics (Deajeon, Korea). Moloney murine leukemia virus (M-MLV) reverse transcriptase was purchased from Invitrogen (Carlsbad, CA, USA). RNAzol B was purchased from Tel-Test (Friendswood, TX, USA).

### 2.3. Sample Collection

AGS is a human, female-origin, EBV-negative gastric adenocarcinoma cell line (KCLB^®^ 21739). The counterpart cell line (AGS-EBV) is a recombinant EBV-infected AGS cell line that was previously described [22,23]. SNU-719 is a male human gastric adenocarcinoma cell line (KCLB^®^00719) naturally infected with EBV and was used as a positive control for EBV infection for EBV in vitro model validation [24]. All cell lines were cultured in RPMI-1640 medium containing 10% fetal bovine serum, 0.25 μg/mL amphotericin B, 100 μg/mL streptomycin, 100 U/mL penicillin, and 25 mM sodium bicarbonate. In the culture of AGS-EBV, EBV-infected cells were selected by adding 400 μg/mL of G418 to the medium. Before analyses, AGS-EBV cells were cultured without G418 for 48 h. All cells were cultured in a 5% CO₂ incubator at 37 °C. For the lipid and metabolite analyses, independently cultured AGS and AGS-EBV cells were counted, and 3 × 10^6^ cells were dispensed into a 1.7 mL microcentrifuge tube. The dispensed cells were centrifuged at 13,000 rpm for 1 min at 4 °C. The supernatant was removed, and the cells were resuspended in sterile distilled water. The cells were then centrifuged at 13,000 rpm for 1 min, and the supernatant was removed. The cell pellets were kept at −80 °C until use.

### 2.4. Sample Preparation for Untargeted Lipidomics

Each extraction solvent included the following lipids: 1.66 μg/mL LPC 17:0, 1.66 μg/mL PC (10:0/10:0), 1.33 μg/mL PE (10:0/10:0), and 3.33 μg/mL SM (18:1/17:0) in methanol and 1.33 μg/mL LPE 17:1, 1 μg/mL Cer (18:1/17:0), 0.8 μg/mL TG (17:0/17:1/17:0, *d*_5_), and 1.66 μg/mL DG in MTBE. The lipid extraction protocol was adapted from the MTBE methodology [25] and modified for in vitro samples. In short, 300 μL of methanol and 250 μL of water were added to each cell pellet, and that sample mixture was vortexed. The tubes were frozen in liquid nitrogen for 5 min and thawed on ice three times. After that, 1000 μL of MTBE was added to the tubes, and every tube was vortexed for 1 h in a thermoshaker at 1500 rpm and 4 °C and then centrifuged at 16,000 rcf for 10 min at 4 °C. Then, 1 mL of the hydrophobic layer was filtered through a hydrophobic syringe filter unit (PTFE/0.2 μm/13 mm) (ADVANTEC^®^, Japan), and the solvent was dried completely by a nitrogen purge at room temperature. The samples were then kept at −80 °C until analysis by ultraperformance liquid chromatography–quadrupole–time of flight mass spectrometry (UPLC-QToF MS).

### 2.5. Sample Preparation for Pseudotargeted and Untargeted Metabolomics

The internal standard (IS) mixtures were constituted by the two metabolites comprised of deuterium form: 1 μg/mL of benzoic acid (2,3,4,5,6-*d*_5_) and fructose (U-^13^C_6_) in 50% methanol (−20 °C). The extraction protocol was obtained from a previous study and optimized to profile the polar metabolites in the in vitro samples [26]. First, 600 μL of IS mixture was added to each cell pellet, and then the sample mixture was vortexed. The tubes were frozen in liquid nitrogen for 5 min and thawed on ice three times. Methanol (900 μL, −80 °C) was added to each tube and incubated for 24 h at −80 °C, followed by centrifugation at 16,000 rcf for 10 min at 4 °C. A 1300 μL aliquot was divided into three: a 550 μL sample for the gas chromatography–mass spectrometry (GC-MS) analysis, a 550 μL sample for the high-performance liquid chromatography coupled with triple quadrupole mass spectrometry (HPLC-QqQ MS) analysis, and 200 μL for the quality control (QC) pool. The collected QC pool was divided into identical 550 μL QC samples. Every sample was evaporated by nitrogen purge at room temperature. For untargeted metabolomics by GC-MS, 40 μL of pyridine containing methoxyamine hydrochloride (15 mg/mL) was added to microcentrifuge tubes for methoxyamination, followed by incubation for 90 min at 30 °C. Then, 40 μL of BSTFA with 1% TMCS was added for trimethylsilylation, and it was incubated for 15 min at 70 °C. At that point, 70 μL of the aliquots was taken for GC-MS analysis after centrifugation. For the large-scale targeted metabolomics by HPLC-QqQ MS, 60 μL of water (4 °C) was added to each tube, followed by centrifugation at 16,000 rcf for 10 min at 4 °C. Then, 50 μL was used for HPLC-QqQ MS data acquisition.

### 2.6. Lipid Analysis by UPLC-QToF MS

The lipid analysis was conducted by UPLC-QToF MS (Agilent 6530 QToF MS coupled with an Agilent 1290 UPLC system; Agilent, Los Angeles, CA, USA). Dried samples were reconstituted with 100 μL of a methanol/toluene mixture (9:1 *v/v*) and then injected into the UPLC system using an Acquity UPLC SCH C18 column (2.1 × 100 mm, 1.7 μm) combined with an Acquity UPLC CSH C18 VanGuard precolumn (2.1 × 5 mm, 1.7 μm) (Waters, Milford, MA) for separation. The gradient conditions and solvents (A: 60:40 (*v/v*) acetonitrile:water and B: 90:10 (*v/v*) isopropanol:acetonitrile with 10 mM ammonium formate and 0.1% formic acid in both mobile phases) were adopted and modified from previous studies [27]. The information was acquired in positive ion mode. The MS condition was established following a previous study using auto MS/MS data-dependent acquisition mode and scan mode with proper modifications [28].

### 2.7. Metabolite Analysis by HPLC-QqQ MS

The pseudotargeted metabolite analysis was conducted by HPLC-QqQ MS (Agilent 6460 coupled with an Agilent 1260 HPLC system, QqQ MS; Agilent, Los Angeles, CA). Dried samples were resuspended with 60 μL of water and then injected into the HPLC system (Agilent, CA, USA) through an Amide XBridge HPLC column (4.6 × 100 mm, 3.5 µm) with a guard column (Waters, Milford, MA, USA) for the separation. The gradient conditions and solvents (A: 95:5 (*v/v*) water:acetonitrile with ammonium acetate (20 mM) and ammonium hydroxide (20 mM) and B: 100% acetonitrile) were adopted and modified [29]. The data were acquired by electrospray ionization (+)/(−) ion-switching targeted metabolomics

### 2.8. Metabolite Analysis by GC-MS

The untargeted metabolite analysis was performed by GC-MS (Shimadzu GCMS-QP2010 system; Shimadzu, Kyoto, Japan). The derivatized samples were injected into the GC-MS system through a DB-5MS capillary column (30 m × 0.25 mm, 0.25 μm; Agilent, PA, USA). For the separation, the system was set in the split mode (1:2 split), and the injector temperature was kept at 300 °C. The primary temperature of the oven for separation was 100 °C. After holding the primary temperature 2 min, it was increased by 10 °C/min until 170 °C and then by 5 °C/min until 300 °C, where it was maintained for 5 min. The temperature of the ion source was 200 °C, and that of the interface was 300 °C. The electron impact mode was used for MS detection, and 70 eV of energy was used for ionization. The mass range scan was from *m/z* 40 to 500.

### 2.9. Data Exploration and Visualization

For the data exploration, we used principal component analysis (PCA) and heatmap, as previously described [15]. We used PCA to visualize the distribution of the samples between comparable groups and detect potential outliers. The heatmap was used to highlight contrasting features (e.g., genes, polar metabolites, or lipids) between comparison groups [30,31].

### 2.10. Survival Analysis

The survival analysis was conducted using KM Plotter with the following settings: patient splitting criterion was median; follow up threshold: all; no restriction about the subtypes or clinical cohorts [32].

### 2.11. Pathway Enrichment Analysis

We conducted a pathway enrichment analysis of dysregulated metabolites using the Kyoto Encyclopedia of Gene and Genomes database integrated into the MetaboAnalyst interface. The pathway enrichment analysis of dysregulated lipids was conducted using LION [33].

### 2.12. Statistical Analysis

For gene expression analysis, limma was applied with the following parameters: specify organism, *Homo sapiens* (human); data type, microarray data (intensities); ID type, Entrez ID; gene-level summarization, median; variance filter, 15; low abundance, 5; filter unannotated genes, check; normalization, quantile; primary factor, class; adjusted *p*-value, 0.05; log_2_-fold change (log_2_FC), 1.0. The analysis was done using NetworkAnalyst [31]. Data normalization and statistical analyses of the data from metabolomics and lipidomics were done using MetaboAnalyst [30]. Data are presented as means and standard deviations unless otherwise specified. In this study, a *p*-value below 0.05 was considered to be statistically significant. For multiple hypothesis testing, we applied an adjusted *p*-value threshold of 0.10 using the Benjamini–Hochberg procedure.

### 2.13. Data Availability

The gene differential expression data were derived from the microarray analysis of five different paired samples from the AGS-EBV and AGS cell lines. Total RNA was extracted by RNAzol B reagents from five separate cultures of AGS and AGS-EBV cells. The microarray analysis comparing AGS and AGS-EBV was conducted by the Sentrix Human-6 v2 Expression BeadChip (deposited as GSE135644). The raw mRNA microarray data from EBVaGC and EBVnGC patients were adopted from a published study (deposited as GSE51575 in GEO).

### 2.14. Quantitative Reverse Transcription–Polymerase Chain Reaction (RT-PCR)

After harvesting the cells, total RNA was isolated by the RNAiso Plus reagent following the manufacturer’s protocols. To synthesize cDNA, 2 μg of total RNA, oligo (dT) primers, and M-MLV reverse transcriptase were used. Real-time RT-PCR was performed with a SYBR green qPCR kit combined with a CFX96 real-time PCR system (Bio-Rad, Hercules, CA, USA). The PCR conditions were as follows: 10 min at 95 °C, followed by 10 s at 95 °C, 30 s at 60 °C, and 30 s at 72 °C (40 cycles). The comparative threshold cycle method was used to calculate relative gene expression, with *GAPDH* as an internal standard. The sequences of the primers used for the *EBNA-1* gene were 5′-AGTCGTCTCCCCTTTGGAAT-3′ and 5′-TCCTCACCCTCATCTCCATC-3′, and those used for the *GAPDH* gene were 5′-ATGGGGAAGGTGAAGGTCG-3′ and 5′-GGGGTCATTGATGGCAACAATA-3′.

## 3. Results

### 3.1. EBVaGC Model Establishment and Validation

In the present study, we used an EBVaGC in vitro model to discover metabolic alterations in GC caused by EBV infection, which we accomplished through omics-based profiling at the metabolic level, followed by statistical and pathway analyses. To mimic the infection of EBV in GC, we adopted a well-established EBVaGC in vitro model from a previous study that had validated the success of EBV infection in a typical GC cell line through biological methods [34]. We also validated the model using real-time RT-PCR for the *EBNA-1* EBV antigen (Figure S1).

### 3.2. Metabolic Associated Genes Are Downregulated in EBVaGC

**Figure 1:**
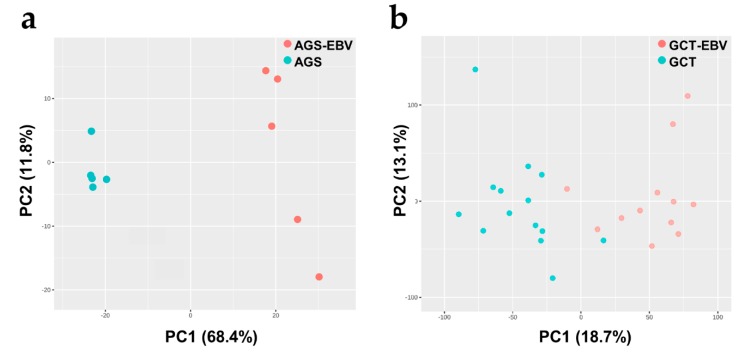
Principal component analysis (PCA) score plots of differences in transcriptome profiles (**a**) between AGS-Epstein–Barr-virus (EBV) and AGS and (**b**) gastric cancer tissue (GCT)-EBV and GCT. Both comparisons reveal clear separations caused by the association of EBV with gastric cancer.

We conducted a microarray-based transcriptomic analysis that compared gene expression between the EBV-positive and EBV-negative gastric carcinoma cell lines. As shown in Figure 1a, the transcriptome profiles of AGS-EBV and AGS were quite different, which again confirmed the unique molecular characteristics of AGS-EBV. The differential expression (DE) analysis showed that 399 genes differed with statistical significance, with the criteria of a log_2_FC and false positive rate (FDR) of 1 and 0.05, respectively. Of those genes, 166 genes were upregulated, and 233 genes were downregulated in AGS-EBV compared with AGS. Interestingly, various genes coding the metabolic enzymes and metabolic regulators were found to be downregulated. For instance, the expression of *LPIN2*, *PCYT2*, *PLA2G16*, *PLA2G4A*, and *PLA2G2A*, which are part of the “glycerophospholipid metabolism”, were significantly diminished in AGS-EBV. Detailed information on these genes and the pathways to which their products belong is presented in Table S1. The gene expression profile of AGS-EBV might have been influenced by the G418 resistance gene introduced to AGS-EBV cells, even though G418 was washed out for 48 h prior to each experiment.

We also conducted a microarray-based transcriptomics analysis to compare the gene expression profiles of the GCT-EBV and GCT clinical samples. The microarray data source for the tissue samples was Kim et al. [35]. As shown in Figure 1b, the GCT-EBV and GCT transcriptome profiles differed, further supporting the distinct molecular signature of EBVaGC. Using the same criteria given above for the gene expression analysis, we found 1548 DE genes, of which 961 were upregulated in GCT-EBV and 587 were downregulated in GCT-EBV. Since we were mainly interested in the effect of EBV infection on EBVaGC, unaffected genes were beyond the scope of investigation. Upregulated genes were not further pursued, as we observed that they were not generally related to metabolic disturbances in EBVaGC.

Overall, 58 metabolic genes in total were found to be downregulated in EBVaGC in either the in vitro or the tissue experiments (Table S1). Those genes belong to a broad spectrum of critical metabolic pathways, such as the biosynthesis of amino acids, fatty acid biosynthesis, central carbon metabolism in cancer, and tryptophan metabolism, and they played a leading role in our downregulated metabolic-pathway-focused analyses. It is worth mentioning that three of the metabolic genes (*CKMT1B*, *ME1*, and *PTGS2*) downregulated in EBVaGC in our analyses that had a significant effect on the survival rate of GC patients were also reported in a previous study [21]. Collectively, the regulation of metabolic genes in EBVaGC differed significantly from that in EBVnGC.

### 3.3. The Effects of Dysregulated Metabolic Regulator Coding Genes on the Survival of GC Patients

**Figure 2:**
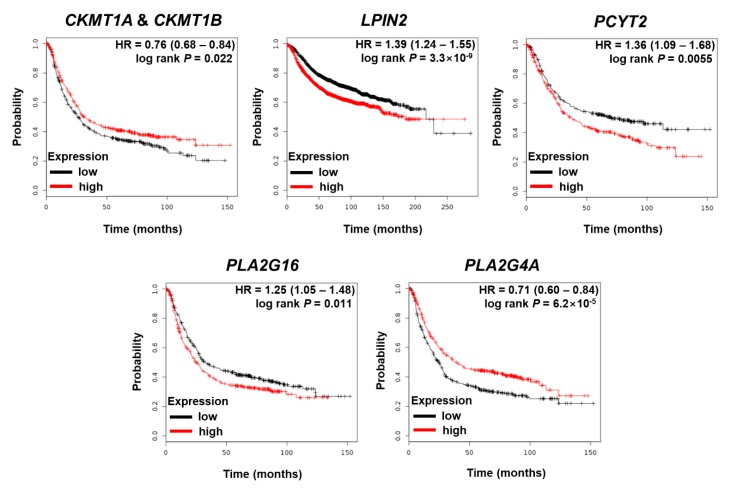
Kaplan–Meier (KM) plots show the effects of the significantly downregulated metabolic genes found in our study. These genes are associated with amino acid and lipid metabolism. HR: hazard ratio.

Based on the 58 significantly downregulated metabolic genes (log_2_FC of 1 and FDR of 0.05), we used the KM estimator to examine the effects of their expression levels on the survival rates of GC patients. As shown in Table S1, 34 metabolic genes associated with EBV altered the hazard ratio (log-rank *p* < 0.05). Thus, EBV’s effects on amino-acid- and lipid-related metabolism might critically alter the survival rate. Among those 34 metabolic genes, 7 genes showed worse outcomes when highly expressed (HR > 1), and 24 genes showed worse outcomes when they were downregulated (HR < 1). The KM plots of genes related to amino acid and lipid metabolism are shown in Figure 2. The other KM plots are given in the Supplementary Materials (Figure S2).

### 3.4. Significant Metabolic Alterations in EBVaGC

**Figure 3:**
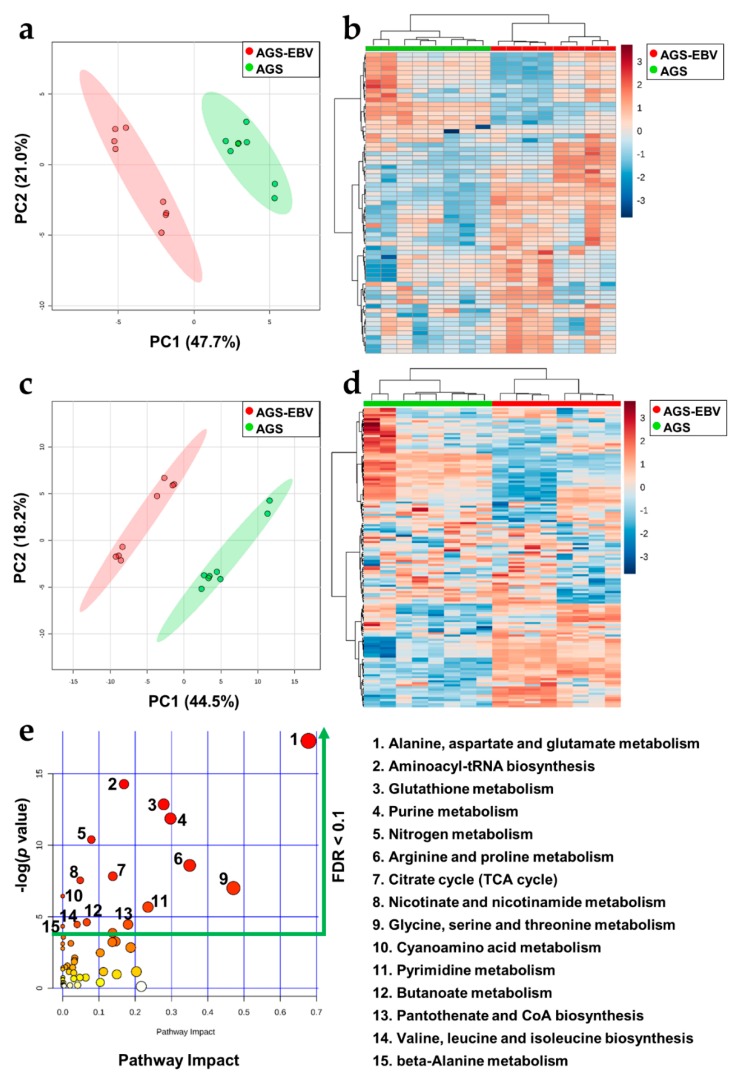
Metabolic profiles of AGS-EBV and AGS made using gas chromatography–mass spectrometry (GC-MS) and high-performance liquid chromatography coupled with triple quadrupole mass spectrometry (HPLC-QqQ MS). (**a**) Two-dimensional (2D) score plot derived using the untargeted GC-MS method showed a tendency of difference between the AGS-EBV and AGS cell lines. (**b**) The heatmap derived from GC-MS-based analysis showed a marked contrast in the expression of the detected metabolites between AGS-EBV and AGS. Also, the (**c**) 2D score plot derived from the HPLC-QqQ MS method and (**d**) heatmap also derived from the large-scale, targeted HPLC-QqQ MS method showed apparent differences between the AGS-EBV and AGS cell lines. (**e**) The pathway analysis based on polar metabolites differentially expressed between AGS-EBV and AGS suggests that 15 metabolic pathways are significantly (*p*-value < 0.05, FDR < 0.1) associated with the effect of EBV infection on gastric cancer.

To more comprehensively investigate the metabolome of EBVaGC, we adopted two strategies for polar metabolite profiling based on the in vitro EBVaGC model. The untargeted metabolomics experiment was conducted using GC-MS. After data alignment, preprocessing, and identification processes, only annotated metabolites were subjected to data normalization and statistical analysis. Our experiment covered 68 metabolites in total. The PCA and heatmap analyses for data exploration and visualization revealed a clear tendency toward different metabolic profiles between AGS-EBV and AGS (Figure 3a,b). When analyzing individual annotated metabolites, we found that 34 metabolites were significantly altered. Among them, 24 were more expressed and 10 were less expressed in AGS-EBV. The LC-MS-based large-scale targeted metabolomics analysis focused mainly on the endogenous metabolites of the biological samples. Similar to the untargeted metabolomics analysis, the targeted analysis revealed a clear separation between AGS-EBV and AGS (Figure 3c,d). In particular, 41 metabolites were more highly expressed and 23 were less expressed in AGS-EBV. From those results, we found 88 metabolites that differed between AGS-EBV and AGS. Those metabolites are associated with 15 metabolic pathways (*p*-value < 0.05, FDR < 0.1), of which “Arginine and proline metabolism”; “Glycine, serine, and threonine metabolism”; and “Alanine, aspartate, and glutamate metabolism” had a high impact (Figure 3e, Table S3). The expression patterns of the detected metabolites were consistent between the two metabolomics platforms.

### 3.5. Significant Lipid Metabolism of EBVaGC

**Figure 4:**
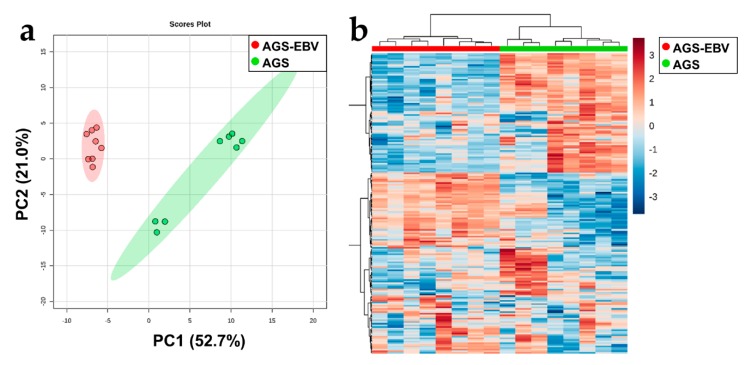
Differential expression of lipids between AGS-EBV and AGS. (**a**) Two-dimensional score plot derived from the lipid profile based on the untargeted UPLC-QToF MS method showed marked differences between the AGS-EBV and AGS cell lines. (**b**) The heatmap showed distinguishable contrast regarding the expression of lipids.

The lipid profiles of EBVaGC and the control were analyzed by our untargeted lipidomics experiment using the UPLC-QToF MS platform. We identified 186 lipids belonging to 15 classes. When we conducted data exploration and visualization using PCA and a heatmap (Figure 4), the lipid profiles between EBVaGC and the control were distinct. Furthermore, we found that 114 lipids were altered significantly, of which 45 were enriched in EBVaGC and 69 were less expressed in EBVaGC. Most of the phospholipids showed increased expression, whereas almost all triacylglycerols decreased. Furthermore, ether-linked lipids also tended to decrease in EBVaGC compared with the control group. The lipid pathway enrichment analysis was performed based on lipids significantly altered by EBV. For the network analysis, the normalized intensity of significantly altered lipids was used with the following parameters: mode, ranking mode; selected local statistics, one-tailed *t*-test (2 conditions); condition of interest, EBV group; control condition, control group. Also, the heatmap module of LION was run using the PCA method with three PCAs. Of the 114 lipids, 101 (88.6%) identifiers could be matched to the LION knowledgebase in both methods. Thirty-seven lipid-related pathways were sorted by the heatmap module analysis, and they belonged to the “physical or chemical properties”, “lipid classification”, “cellular component”, and “function” categories. Based on the heatmap, lateral diffusion was increased, and bilayer thickness was decreased in EBVaGC. Furthermore, ether-linked lipids, sphingolipids, and glycerolipids were downregulated by EBV (Figure S3). Among them, the 15 pathways showed significant alteration (*p*-value ≤ 0.05, FDR < 0.1) by EBV association and were usually related to physical or chemical properties and a specific lipid class change (Table 1).

### 3.6. The Metabolic Landscape of EBVaGC

**Figure 5:**
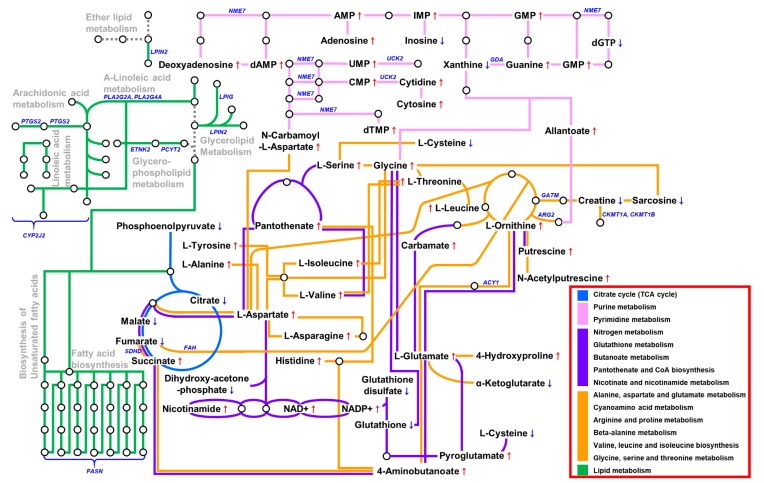
The metabolic landscape of EBV-associated gastric carcinoma.

Combining the results from the transcriptomics, metabolomics, and lipidomics experiments enabled us to construct a putative metabolic landscape for EBVaGC (Figure 5). As shown in Figure 3e and Table 1, a variable number of pathways were affected. The genes related to metabolic pathways were downregulated, as judged from the in vivo and in vitro data. These genes were usually associated with amino acid and lipid metabolisms. Pathway enrichment analyses using polar metabolites demonstrated six pathways related to amino acid metabolism (Figure 3e). In addition, some core genes in amino acid metabolism (*ALDH2*, *CKMT1A*, *CKMT1B*, *DDC*, *FAH*, *GATM*, *MAOA*, and *PNMT*) that decreased in EBVaGC correlated with the metabolomics data. Furthermore, EBVaGC showed apparent differences in physical or chemical properties derived from lipid alterations, such as lateral diffusion and bilayer thickness and the composition of the lipid classes (Table 1). These alterations are related to the genes (*CYP2J2*, *LPIN2*, *PCYT2*, *PLA2G16*, *PLA2G2A*, *PLA2G4A*, and *PTGS2*) that affect lipid metabolism or one of several fatty acid metabolisms.

## 4. Discussion

Molecular subtyping is an essential step for the stratification of patients and the selection of proper clinical interventions. A recent investigation using the proteo-genomics approach suggested that diffuse GCs in young populations include four subtypes: proliferation, immune response, metabolism, and invasion [36]. Comprehensive analyses of the TCGA cohort revealed a strong association between cellular metabolism, including energy production, and EBVaGC—a subtype of GC [13,21]. Because EBVaGC is an important subtype of GC that makes up approximately 5–10% of all cases, determining the regulators associated with the progression of the disease is of great interest. In fact, the immune invasion of EBV in GC is considered to be a potential biomarker for the response of patients to immunotherapy and appears to have clinical benefits [5,37]. In this study, we used post-analysis transcriptomics and metabolomics–lipidomics data to demonstrate that metabolic aberrance is a critical signature of EBVaGC.

We constructed a model of the metabolic landscape at the transcriptomics and metabolomics–lipidomics levels (Figure 5). A total of 58 enzymes and metabolic coding genes were found to be downregulated in EBVaGC. They belong to the central carbon metabolism, amino acid metabolism, and fatty acid metabolism categories, to name a few. In the lipidomics analysis, 114 lipids were confirmed to be altered in the EBVaGC samples. We used two metabolomics strategies and found 88 metabolites that differed significantly between EBVaGC and EBVnGC. Noticeably, we found a clear correlation between the dysregulation of gene expression and the alteration of the metabolome. For instance, EBV appears to alter amino acid metabolism, the TCA cycle, and nucleotide synthesis. Furthermore, aberrant lipid metabolism derived from the lipidomics approach demonstrated that EBVaGC has phospholipid and triacylglycerol expression patterns that differ from those of EBVnGC. Given that lipids are major components of cell membrane structure, these alterations could reconstruct lipid classifications and probably modify several physical or chemical properties.

Amino acids play an essential role in metabolic homeostasis, the organization of body structure, and chemical reaction controls [38]. For instance, amino acids are associated with aberrant homeostasis of reactive oxygen species [39] and cell proliferation in cancer [40]. Nonessential amino acids, such as glutamine, glutamate, glycine, and aspartate, were also reported to contribute to the pathology of cancer [41]. In GC, metabolic reprogramming of the amino acid metabolism altered several amino acid concentrations and upregulated an amino acid transporter gene [42]. In the present study, we found that all amino acids except cysteine were overexpressed in EBVaGC compared with EBVnGC. Seven pathways related to amino acids were found to be significantly altered in the functional analysis: ”glycine, serine, and threonine metabolism”; ”alanine, aspartate, and glutamate metabolism”; ”arginine and proline metabolism”; ”cyanoamino acid metabolism”; ”valine, leucine, and isoleucine biosynthesis”; ”aminoacyl-tRNA biosynthesis”; and ”beta-alanine metabolism.” Although EBV’s effect on the metabolic regulation of GC has not yet been well established, some previous studies about the metabolic regulation of GC mentioned that glycine, serine, valine, phenylalanine, tryptophan, and proline were significantly overexpressed in GC tissue specimens [43]. Another study also reported that aspartate and glutamate levels were increased in GC [44]. The overexpression of amino acids could be associated with the progression of EBVaGC. Also, aberrant expression of amino acids could affect survival phenotypes, which are related to alterations in the genes that code for amino acid metabolism. For instance, less expression of *CKMT1A* and *CKMT1B*, which are involved in arginine and proline metabolism, corresponded with a worse survival rate (Figure 2). Furthermore, our data suggest that metabolites involved in purine/pyrimidine pathways are associated with EBV in GC. It has been reported that concentrations of purine metabolites increase in cancer [45]. Likewise, purinosome, which is a recently identified component of purine metabolism, has a high correlation with the cell cycle [46]. Enzymes connected to pyrimidine nucleotide synthesis were reported to be upregulated in poorly differentiated GC compared with well-differentiated GC and normal tissues [47]. In agreement with previous findings, almost every nucleotide-related metabolite was increased in EBVaGC in our results. Notably, the expression of xanthine decreased in EBVaGC. This pattern corresponds to a previous study, which reported that xanthine oxidoreductase (oxidizes hypoxanthine to xanthine) was downregulated in a group of GC patients with a poor prognosis [48]. Furthermore, among significantly downregulated metabolic genes related to the purine/pyrimidine synthesis pathway, *GDA* downregulation could be associated with a worse outcome (Figure S2).

Although several lines of evidence have suggested that altered lipid metabolism plays a role in EBVaGC progression, high-throughput lipid profiling of EBVaGC has not been clearly reported. Aberrant lipid metabolism plays an important role in carcinogenesis and development [49], and lipid reprogramming is also triggered by tumors. Lipid reprogramming is also involved in phenomena such as tumor progression and membrane homeostasis [50]. In this study, we analyzed three dominant lipid species: phospholipids, sphingolipids, and neutral lipids. We found that TG was downregulated, whereas PC and PE were upregulated in EBVaGC. Interestingly, ether-linked lipids showed relatively low intensities in EBVaGC, even though they are also phospholipid species. Ceramide, which is the backbone of the sphingolipids, seems to be downregulated by EBV infection. Our data suggest that lipid alterations caused by EBV in GC are associated with a low transition temperature and low bilayer thickness [51,52]. Previous studies have shown that the p7 protein of hepatitis C virus (HCV), which oligomerizes in the membrane to form cation channels, substantially thins the surrounding lipid bilayer [53]. The decrease in the bilayer thickness observed in HCV-infected cells is due to the physical change of the bilayer following insertion of viral protein. In contrast, bilayer thickness in EBVaGC is predicted to be reduced mainly due to the change of lipid composition.

EBVaGC demonstrated upregulation of various lipid classes, including diacylglycerophosphocholines and diacylglycerophosphoethanolamines, compared with EBVnGC. Furthermore, ether-linked lipid metabolisms (“contains ether-bond”, “1-alkyl, 2-acylglycerophosphoethanolamines”, and “1-alkyl, 2-acylglycerophosphocholines”) were decreased in EBVaGC (Figure S3). We found a strong correlation between these lipid metabolic alterations and enzymes and lipid-regulator-coding genes. For instance, the expression of *CYP2J2*, *PLA2G2A PLA2G4A*, and *PTGS2* correlated with unsaturated fatty acids, including linoleic acid, α-linoleic acid, and arachidonic acid. We speculate that alteration of unsaturated fatty acid composition and fatty acid elongation affects the physical and chemical properties of lipids. Previous studies reported the overexpression of fatty acid synthase (FASN) in cancer [54,55]. In addition to the length of the fatty acid, the degree of unsaturation of fatty acid was also considered to affect the growth of tumor cells [56]. Furthermore, *LIPG*, *LPIN2*, *ETNK2*, and *PCYT2* regulate glycerolipid and glycerophospholipid metabolism, which contribute to the altered PC, PE, and TG expression found in EBVaGC. Most of all, *LPIN2*, which controls ether lipid metabolism, was decreased in EBVaGC.

The dysregulation of metabolic regulators found in our study appears to be associated with the prognosis of GC patients in a complicated manner. For example, the overexpression of *CKMT1A* and *CKMT1B,* which are the regulators of arginine and proline metabolism, are associated with better survival in GC (Figure 2). *CKMT1A* and *CKMT1B* were found to be downregulated in EBVaGC (Table S1), which could contribute to the progression of EBVaGC. Also, *FASN* and *PLA2G4A,* which regulate fatty acid biosynthesis, were decreased in EBVaGC (Table S1), which could lead to worse survival of GC patients. The downregulation of those two genes might affect the tumorigenesis of EBVaGC. Collectively, even though EBVaGC is known to be less aggressive than other subtypes of GC [9,10], EBV appears to profoundly reprogram the metabolism of GC via the aberrant expression of various lipids and polar metabolites. The opposite effects from the downregulation of metabolic regulators suggest that EBV causes a sophisticated metabolic rewire in GC. As EBV expresses very limited EBV genes such as *EBNA-1, LMP2A, EBERs*, and *BART* miRNAs in EBVaGC [22], it is of great interest to understand how the dramatic metabolic landscape changes observed in our results are possible. Further investigations are needed to explore the underlying mechanisms of this multifaceted phenomenon.

Our study has several limitations that need to be stated. Although the postanalysis multi-omics integration is useful for generating new hypotheses, it did not allow us to capture all possible associations among the different layers of omics data. For example, several glycan-related pathways involving *FUT4*, *B4GALT1*, and *GCNT3* showed a substantial alteration in EBVaGC in the gene expression analysis, but no accompanying metabolites were found (Figure S3). Because we focused on the metabolic-related transcriptome, metabolome, and lipidome to discover the effect of EBV in GC within the metabolic network, our data do not fully cover all the biological processes happening during the tumorigenesis of GC. Although both in vitro and clinical samples were analyzed for transcriptomics data in this study, metabolomics and lipidomics data were obtained using only the in vitro model. Thus, the exact mechanisms by which the metabolic dysregulation we found contributes to the progression of EBVaGC remain to be elucidated by follow-up studies.

## 5. Conclusions

By using a comprehensive multi-omics approach, we established the metabolic landscape of EBVaGC and generated a comprehensive framework for mechanistic studies. Central carbon metabolism, amino acid metabolism, and glycerophospholipid and triacylglycerol metabolism were significantly altered in EBVaGC compared with EBVnGC. The metabolic dysregulations in EBVaGC could be associated with the clinical prognosis of GC patients. Although the prognostic tendency in the metabolic genes related to EBVaGC are the opposite of those found in previous studies on the systemic scale, the expression pattern of polar metabolites correlated well with preceding studies. Also, differences in lipid expression between EBVaGC and EBVnGC were notably supported in the present study. Our results suggest that EBV infection causes the metabolic reprogramming of cancer cells, and those metabolic alterations could modulate the progression of EBVaGC cooperatively with other biological events. Collectively, these findings could be used to discover and validate new biomarkers and therapeutics for EBVaGC.

## Figures and Tables

**Table 1:** The lipid metabolic pathways derived from lipids significantly altered by EBV.

	LION ID	Pathways	Annotated	*p*-Value	FDR^*^
1	LION:0080973	Below average bilayer thickness	22	3.50 × 10^−10^	1.67 × 10^−8^
2	LION:0001741	Below average transition temperature	24	6.30 × 10^−10^	1.67 × 10^−8^
3	LION:0080982	Above average lateral diffusion	21	1.60 × 10^−9^	2.83 × 10^−8^
4	LION:0080968	Very low bilayer thickness	15	1.80 × 10^−6^	1.91 × 10^−6^
5	LION:0080980	Very high lateral diffusion	15	5.10 × 10^−6^	1.91 × 10^−6^
6	LION:0001735	Very low transition temperature	12	3.50 × 10^−10^	4.51 × 10^−5^
7	LION:0000030	Diacylglycerophosphocholines	20	2.00 × 10^−4^	1.51 × 10^−3^
8	LION:0080979	High lateral diffusion	8	6.90 × 10^−4^	4.12 × 10^−3^
9	LION:0001736	Low transition temperature	12	7.00 × 10^−4^	4.12 × 10^−3^
10	LION:0012010	Membrane component	63	9.70 × 10^−4^	5.14 × 10^−3^
11	LION:0080969	Low bilayer thickness	7	2.58 × 10^−3^	5.14 × 10^−3^
12	LION:0000095	Headgroup with positive charge/zwitter ion	61	3.94 × 10^−3^	1.74 × 10^−2^
13	LION:0000084	Ceramide phosphocholines (sphingomyelins)	8	0.012	4.86 × 10^−2^
14	LION:0000038	Diacylglycerophosphoethanolamines	9	0.015	5.69 × 10^−2^
15	LION:0000465	Neutral intrinsic curvature	33	0.022	7.93 × 10^−2^

^*^ FDR: false discovery rate.

## Supplementary Materials

The following are available online at https://www.mdpi.com/2073-4409/8/10/1220/s1, Table S1: Downregulated genes that code metabolic enzymes and metabolic regulators in EBVaGC, Table S2: Polar metabolites that differed significantly between AGS-EBV and AGS cells, Table S3: Pathway analysis based on differences in polar metabolites between EBVaGC and EBVnGC, Figure S1: Real-time RT-PCR to validate the in vitro EBVaGC model. Figure S2: Kaplan–Meier survival plots of gastric cancer (GC) for metabolic genes significantly downregulated in EBVaGC, Figure S3: Heatmap of pathway enrichment analysis based on lipids significantly altered in EBVaGC, Figure S4: Metabolic pathways with transcripts significantly downregulated and metabolites altered by EBV infection in gastric cancer.
