# Supplementary material for: Comprehensive Multi-Omics Analysis Reveals Aberrant Metabolism of Epstein–Barr-Virus-Associated Gastric Carcinoma
Source: Cells. 2019 Oct 8;8(10):1220. doi: 10.3390/cells8101220 (PMC6829863; doi:10.3390/cells8101220)
Supplement: Supplementary file 1 [file cells-08-01220-s001.pdf]

<Supplementary Materials>

Comprehensive Multi-Omics Analysis Reveals Aberrant Metabolism of Epstein–Barr-Virus-Associated Gastric Carcinoma

Sang Jun Yoon [1,†], Jun Yeob Kim [2,†], Nguyen Phuoc Long [1], Jung Eun Min [1], Hyung Min Kim [1], Jae Hee Yoon [2], Nguyen Hoang Anh [1], Myung Chan Park [2], Sung Won Kwon [1,*] and Suk Kyeong Lee [2,*]

[1] College of Pharmacy, Seoul National University, Seoul 08826, Korea; supercanboy@snu.ac.kr (S.J.Y.); phuoclong@snu.ac.kr (N.P.L.); mje0107@snu.ac.kr (J.E.M.) snuhmkim04@snu.ac.kr (H.M.K.); 2018-23140@snu.ac.kr (N.H.A.)

[2] Department of Medical Life Sciences, Department of Biomedicine & Health Sciences, College of Medicine, The Catholic University of Korea, Seoul 06591, Korea; kjygod1@catholic.ac.kr (J.Y.K.); yjh823@catholic.ac.kr (J.H.Y.); pmcreg@catholic.ac.kr (M.C.P.)

* Correspondence: sukklee@catholic.ac.kr (S.K.L.); swkwon@snu.ac.kr (S.W.K.); Tel.: +82-0-2258-7480 (S.K.L); Tel.: +82-2-880-7844 (S.W.K.); Fax.: +82-504-201-2396 (S.K.L.); Fax.: +82-2-886-7844 (S.W.K.)

† These authors contributed equally to this work.

Received: date; Accepted: date; Published: date

Table of Contents

Table S1: Downregulated genes that code metabolic enzymes and metabolic regulators in EBVaGC.

Table S2: Polar metabolites that differed significantly between AGS-EBV and AGS cells.

Table S3: Pathway analysis based on differences in polar metabolites between EBVaGC and EBVnGC.

Figure S1: Real-time RT-PCR to validate the *in vitro* EBVaGC model.

Figure S2: Kaplan–Meier survival plots of gastric cancer (GC) for metabolic genes significantly downregulated in EBVaGC.

Figure S3: Heatmap of pathway enrichment analysis based on lipids significantly altered in EBVaGC.

Figure S4: Metabolic pathways with transcripts significantly downregulated and metabolites altered by EBV infection in gastric cancer.

Table S1. Downregulated genes that code metabolic enzymes and metabolic regulators in EBVaGC.

	Gene ID	Origin of the Gene	Related metabolism	*p*-value
1	*ACACA**	Tissue	"Metabolic pathways"	1.39×10^{-5}
2	*ACOT1**	Cell line	"Metabolic pathways"	5.35×10^{-13}
3	*ACOT2**	Cell line	"Metabolic pathways"	2.67×10^{-9}
4	*ACY1*	Tissue	"Metabolic pathways"	1.46×10^{-4}
5	*ALDH1A1**	Cell line	"Metabolic pathways"	4.84×10^{-11}
6	*ALDH2*	Cell line	"Metabolic pathways," "Arginine and proline metabolism," "Ascorbate and aldarate metabolism," "Tryptophan metabolism"	2.61×10^{-6}
7	*ALDOC*	Tissue	"Metabolic pathways," "Fructose and mannose metabolism," "Pentose phosphate pathway"	1.93×10^{-5}
8	*ALG9*	Cell line	"Metabolic pathways"	1.56×10^{-11}
9	*ARG2**	Tissue	"Metabolic pathways"	3.63×10^{-5}
10	*B4GALNT1**	Tissue	"Metabolic pathways"	1.66×10^{-4}
11	*B4GALNT4**	Tissue	"Metabolic pathways"	2.38×10^{-6}
12	*CKMT1A**	Cell line	"Metabolic pathways," "Arginine and proline metabolism"	9.98×10^{-9}
13	*CKMT1B**	Cell line	"Metabolic pathways," "Arginine and proline metabolism"	3.81×10^{-9}
14	*CYP2J2*	Cell line	"Metabolic pathways," "Linoleic acid metabolism," "Arachidonic acid metabolism"	1.74×10^{-12}
15	*DDC*	Cell line	"Metabolic pathways," "Tryptophan metabolism," "Phenylalanine metabolism"	1.60×10^{-9}
16	*DHCR24*	Cell line	"Metabolic pathways"	3.15×10^{-7}
17	*ETNK2**	Tissue	"Metabolic pathways"	1.44×10^{-4}
18	*EXT1**	Cell line	"Metabolic pathways"	6.92×10^{-11}
19	*FAH**	Tissue	"Metabolic pathways"	2.15×10^{-7}
20	*FASN**	Tissue	"Metabolic pathways"	8.81×10^{-7}
21	*FUT4**	Cell line	"Metabolic pathways"	2.40×10^{-12}
22	*GALC**	Cell line	"Metabolic pathways"	9.37×10^{-16}
23	*GALNT5*	Cell line	"Metabolic pathways"	2.38×10^{-10}
24	*GALNT6*	Cell line	"Metabolic pathways"	1.56×10^{-11}
25	*GATM*	Cell line	"Metabolic pathways," "Arginine and proline metabolism"	5.27×10^{-10}

26	*GCNT3*	Cell line	"Metabolic pathways"	4.12×10^{-4}
27	*GDA**	Cell line	"Metabolic pathways"	5.70×10^{-12}
28	*HMOX1*	Cell line	"Metabolic pathways"	4.62×10^{-9}
29	*HSD17B2**	Cell line	"Metabolic pathways"	1.22×10^{-9}
30	*HSD17B7**	Tissue	"Metabolic pathways"	1.28×10^{-4}
31	*HSD3B1**	Cell line	"Metabolic pathways"	1.23×10^{-10}
32	*IMPAD1*	Cell line	"Metabolic pathways"	8.05×10^{-6}
33	*ISYNA1*	Tissue	"Metabolic pathways"	8.63×10^{-5}
34	*LIPG**	Cell line	"Metabolic pathways"	1.33×10^{-10}
35	*LPIN2**	Cell line	"Metabolic pathways," "Glycerophospholipid metabolism"	2.19×10^{-8}
36	*MAOA*	Cell line	"Metabolic pathways," "Arginine and proline metabolism," "Tryptophan metabolism," "Phenylalanine metabolism"	3.47×10^{-6}
37	*ME1**	Tissue	"Metabolic pathways"	1.07×10^{-4}
38	*MGAT4A**	Cell line	"Metabolic pathways"	5.62×10^{-9}
39	*NAGS**	Tissue	"Metabolic pathways"	9.01×10^{-5}
40	*NME7*	Tissue	"Metabolic pathways"	3.24×10^{-5}
41	*PCYT2**	Cell line	"Metabolic pathways," "Glycerophospholipid metabolism"	2.80×10^{-6}
42	*PFKM**	Tissue	"Metabolic pathways," "Fructose and mannose metabolism," "Pentose phosphate pathway"	1.69×10^{-4}
43	*PGM1*	Cell line	"Metabolic pathways," "Amino sugar and nucleotide sugar metabolism"	1.88×10^{-11}
44	*PLA2G16**	Cell line	"Metabolic pathways," "Linoleic acid metabolism," "Arachidonic acid metabolism," "alpha-Linolenic acid metabolism," "Glycerophospholipid metabolism," "Ether lipid metabolism"	9.11×10^{-8}
45	*PLA2G2A*	Cell line	"Metabolic pathways," "Linoleic acid metabolism," "Arachidonic acid metabolism," "alpha-Linolenic acid metabolism," "Glycerophospholipid metabolism," "Ether lipid metabolism"	7.10×10^{-8}
46	*PLA2G4A**	Cell line	"Metabolic pathways," "Linoleic acid metabolism," "Arachidonic acid metabolism," "alpha-Linolenic acid metabolism," "Glycerophospholipid metabolism," "Ether lipid metabolism"	2.20×10^{-7}
47	*PNMT*	Tissue	"Metabolic pathways," "Tyrosine metabolism"	7.77×10^{-7}
48	*PTGS2**	Cell line	"Metabolic pathways," "Arachidonic acid metabolism"	1.71×10^{-8}
49	*PYCRL**	Tissue	"Metabolic pathways"	8.91×10^{-5}
50	*RENBP*	Cell line	"Metabolic pathways," "Amino sugar and nucleotide sugar metabolism"	2.94×10^{-7}
51	*RGN**	Cell line	"Metabolic pathways," "Ascorbate and aldarate metabolism"	2.62×10^{-14}
52	*SDHD*	Cell line	"Metabolic pathways"	3.68×10^{-7}

53	*SQLE**	Tissue	"Metabolic pathways"	1.92×10^{-4}
54	*ST6GALNAC3**	Cell line	"Metabolic pathways"	5.53×10^{-10}
55	*UAP1L1**	Cell line	"Metabolic pathways," "Amino sugar and nucleotide sugar metabolism"	4.65×10^{-6}
56	*UCK2*	Tissue	"Metabolic pathways"	2.04×10^{-5}
57	*UGT1A6*	Cell line	"Metabolic pathways," "Ascorbate and aldarate metabolism"	2.92×10^{-14}
58	*XYLB*	Tissue	"Metabolic pathways"	1.12×10^{-4}

* Genes that significantly affect the survival rate of gastric cancer patients.

Table S2. Polar metabolites that differed significantly between AGS-EBV and AGS cells (*P*-value ≤ 0.05, FDR < 0.1). ▲: up-regulated in EBVaGC compared to control; ▼: down-regulated in EBVaGC compared to control.

	Metabolites	KEGG ID	HMDB ID	Expression	Platform
1	2-Aminomalonic acid	C00872	HMDB0001147	▲	GC
2	4-Aminobutanoic acid	C00334	HMDB0000112	▲	LC/GC
3	4-Hydroxyproline	C01157	HMDB0000725	▲	LC
4	Adenosine	C00212	HMDB0000050	▲	LC
5	Allantoate	C00499	HMDB0001209	▲	LC
6	AMP	C00020	HMDB0000045	▲	LC
7	Arachidonic acid	C00219	HMDB0001043	▲	GC
8	Biotin	C00120	HMDB0000030	▲	LC
9	Boric acid	C12486	HMDB0035731	▲	GC
10	carbamate	C01563	HMDB0003551	▲	GC
11	Carnitine	C00487	HMDB0000062	▼	LC
12	Cholesterol	C00187	HMDB0000067	▼	GC
13	Cholesteryl sulfate	C18043	HMDB0000653	▼	LC
14	Citric acid	C00158	HMDB0000094	▼	LC
15	CMP	C00055	HMDB0000095	▲	LC
16	Creatine	C00300	HMDB0000064	▼	LC
17	Creatinine	C00300	HMDB0000064	▼	LC/GC
18	Cytidine	C00475	HMDB0000089	▲	LC
19	Cytosine	C00380	HMDB0000630	▲	LC
20	dAMP	C00360	HMDB0000905	▲	LC
21	Deoxyadenosine	C00559	HMDB0000101	▲	LC
22	D-Erythrotetrofuranose			▼	GC
23	dGMP	C00362	HMDB0001044	▲	LC
24	dGTP	C00286	HMDB0001440	▼	LC
25	Dihydroxy-acetone-phosphate	C00111	HMDB0001473	▼	LC
26	D-Lactose	C00243	HMDB0000186	▼	GC
27	dTMP	C00364	HMDB0001227	▲	LC
28	Ethanolamine	C00189	HMDB0000149	▲	LC
29	FAD	C00189	HMDB0000149	▼	LC
30	Folate	C00504	HMDB0000121	▲	LC
31	Fructose-1,6-bisphosphate	C05378	HMDB0001058	▼	LC
32	Fumaric acid	C00122	HMDB0000134	▼	GC
33	Geranyl-PP	C00341	HMDB0001285	▲	LC
34	Glutathione	C00051	HMDB0000125	▼	LC
35	Glutathione disulfide	C00127	HMDB0003337	▼	LC
36	Glycerophosphocholine	C00670	HMDB0000086	▲	LC
37	GMP	C00144	HMDB0001397	▲	LC
38	Guanine	C00242	HMDB0000132	▲	LC
39	Hexanoic acid	C01585	HMDB0000535	▲	GC
40	Histidine	C00135	HMDB0000177	▲	LC
41	Imidazoleacetic acid	C02835	HMDB0002024	▼	LC
42	IMP	C00130	HMDB0000175	▲	LC
43	Inosine	C00294	HMDB0000195	▼	LC
44	Lactic Acid	C01432	HMDB0000190	▼	LC/GC
45	L-Alanine	C00041	HMDB0000161	▲	LC/GC
46	L-Asparagine	C00152	HMDB0000168	▲	GC
47	L-Aspartate	C00049	HMDB0000191	▲	LC/GC
48	L-Cysteine	C00097	HMDB0000574	▼	GC
49	L-Glutamate	C00025	HMDB0000148	▲	LC/GC
50	L-Glycine	C00037	HMDB0000123	▲	LC/GC
51	L-isoleucine	C00407	HMDB0000172	▲	LC/GC
52	L-Leucine	C00123	HMDB0000687	▲	LC/GC
53	L-Serine	C00065	HMDB0000187	▲	GC
54	L-Threonine	C00188	HMDB0000167	▲	LC
55	L-Tyrosine	C00082	HMDB0000158	▲	GC
56	L-Valine	C00183	HMDB0000883	▲	GC

57	Maleic acid	C01384	HMDB0000176	▼	LC
58	Malic acid	C00149	HMDB0000156	▼	GC
59	Methionine sulfoxide	C02989	HMDB0002005	▲	LC
60	Methylcysteine		HMDB0002108	▲	LC
61	Myo-Inositol	C00137	HMDB0000211	▲	GC
62	N-Acetylputrescine	C02714	HMDB0002064	▲	LC
63	NAD+	C00003	HMDB0000902	▲	LC
64	NADH	C00004	HMDB0001487	▲	LC
65	NADP+	C00006	HMDB0000217	▲	LC
66	N-carbamoyl-L-aspartate	C00438	HMDB0000828	▲	LC
67	Nicotinamide	C00153	HMDB0001406	▲	LC
68	Oleic Acid	C00712	HMDB0000207	▲	GC
69	Ornithine	C00077	HMDB0000214	▼	LC
70	Palmitelaidic acid		HMDB0012328	▲	GC
71	Pantothenic acid	C00864	HMDB0000210	▲	LC/GC
72	Phosphoenolpyruvate	C00074	HMDB0000263	▼	LC
73	Phosphoric acid	C00009	HMDB0001429	▼	GC
74	Phosphorylethanolamine	C00346	HMDB0000224	▲	GC
75	p-Hydroxybenzoate	C00156	HMDB0000500	▼	LC
76	Pipecolic acid	C00408	HMDB0000070	▼	LC
77	Putrescine	C00134	HMDB0001414	▲	GC
78	Pyridoxine	C00314	HMDB0000239	▼	LC
79	Pyroglutamic acid	C01879	HMDB0000267	▲	GC
80	Pyrophosphate	C00013	HMDB0000250	▼	GC
81	Sarcosine	C00213	HMDB0000271	▼	LC
82	Shikimate-3-phosphate	C03175		▲	LC
83	Succinate/Methylmalonic acid	C02170	HMDB0000202	▲	LC
84	Succinic acid	C00042	HMDB0000254	▲	GC
85	Trehalose-6-Phosphate	C00689	HMDB0001124	▲	LC
86	UMP	C00105	HMDB0000288	▲	LC
87	Xanthine	C00385	HMDB0000292	▼	LC
88	α-Ketoglutarate	C00940	HMDB0001552	▼	LC

Table S3. Pathway analysis based on differences in polar metabolites between EBVaGC and EBVnGC.

	Pathway name	Total	Expected	Hits	Raw *p*-value	FDR	Impact
1	Alanine, aspartate, and glutamate metabolism	24	0.81	9	3.07×10^{-8}	2.46×10^{-6}	0.67806
2	Aminoacyl-tRNA biosynthesis	75	2.52	13	6.32×10^{-7}	2.53×10^{-5}	0.16902
3	Glutathione metabolism	38	1.28	9	2.61×10^{-6}	6.95×10^{-5}	0.27861
4	Purine metabolism	92	3.10	13	7.03×10^{-6}	1.41×10^{-4}	0.2973
5	Nitrogen metabolism	39	1.31	8	3.06×10^{-5}	4.90×10^{-4}	0.07895
6	Arginine and proline metabolism	77	2.59	10	1.86×10^{-4}	2.48×10^{-3}	0.35026
7	Citrate cycle (TCA cycle)	20	0.67	5	3.97×10^{-4}	4.54×10^{-3}	0.13818
8	Nicotinate and nicotinamide metabolism	44	1.48	7	5.27×10^{-4}	5.27×10^{-3}	0.0482
9	Glycine, serine, and threonine metabolism	48	1.62	7	9.08×10^{-4}	8.07×10^{-3}	0.47083
10	Cyanoamino acid metabolism	16	0.54	4	1.59×10^{-3}	0.013	0
11	Pyrimidine metabolism	60	2.02	7	3.44×10^{-3}	0.025	0.23504
12	Butanoate metabolism	40	1.35	5	9.95×10^{-3}	0.066	0.0663
13	Valine, leucine, and isoleucine biosynthesis	27	0.91	4	0.012	0.066	0.03975
14	Pantothenate and CoA biosynthesis	27	0.91	4	0.012	0.066	0.18014
15	beta-Alanine metabolism	28	0.91	4	0.013	0.070	0

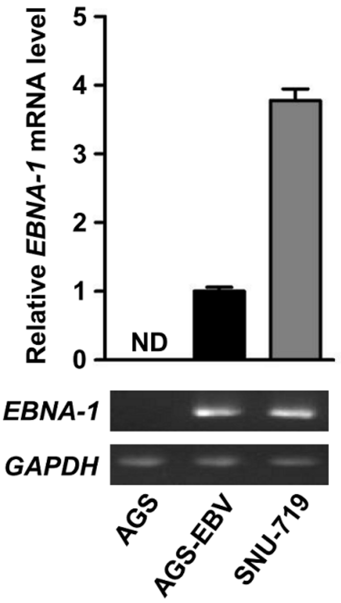

Figure S1. Real-time RT-PCR to validate the *in vitro* EBVaGC model. Real-time RT-PCR analysis of *EBNA-1* mRNA was carried out using a SYBR green qPCR kit. SNU-719, a gastric cancer cell line naturally infected with EBV, was used as a positive control for EBV infection. *EBNA-1* mRNA was chosen to confirm EBV infection because it is known to be expressed in all EBV-infected cells. *GAPDH* was used as an internal control. Similar results were obtained in two additional independent real-time RT-PCR experiments, and the means +/- SD from all three experiments are presented in the upper panel (n = 3). After the real-time RT-PCR reaction, the PCR products were electrophoresed on a 2% agarose gel to visualize the amplicons, as shown in the lower panel. For AGS cells, we could not detect *EBNA-1* expression. ND = not detected.

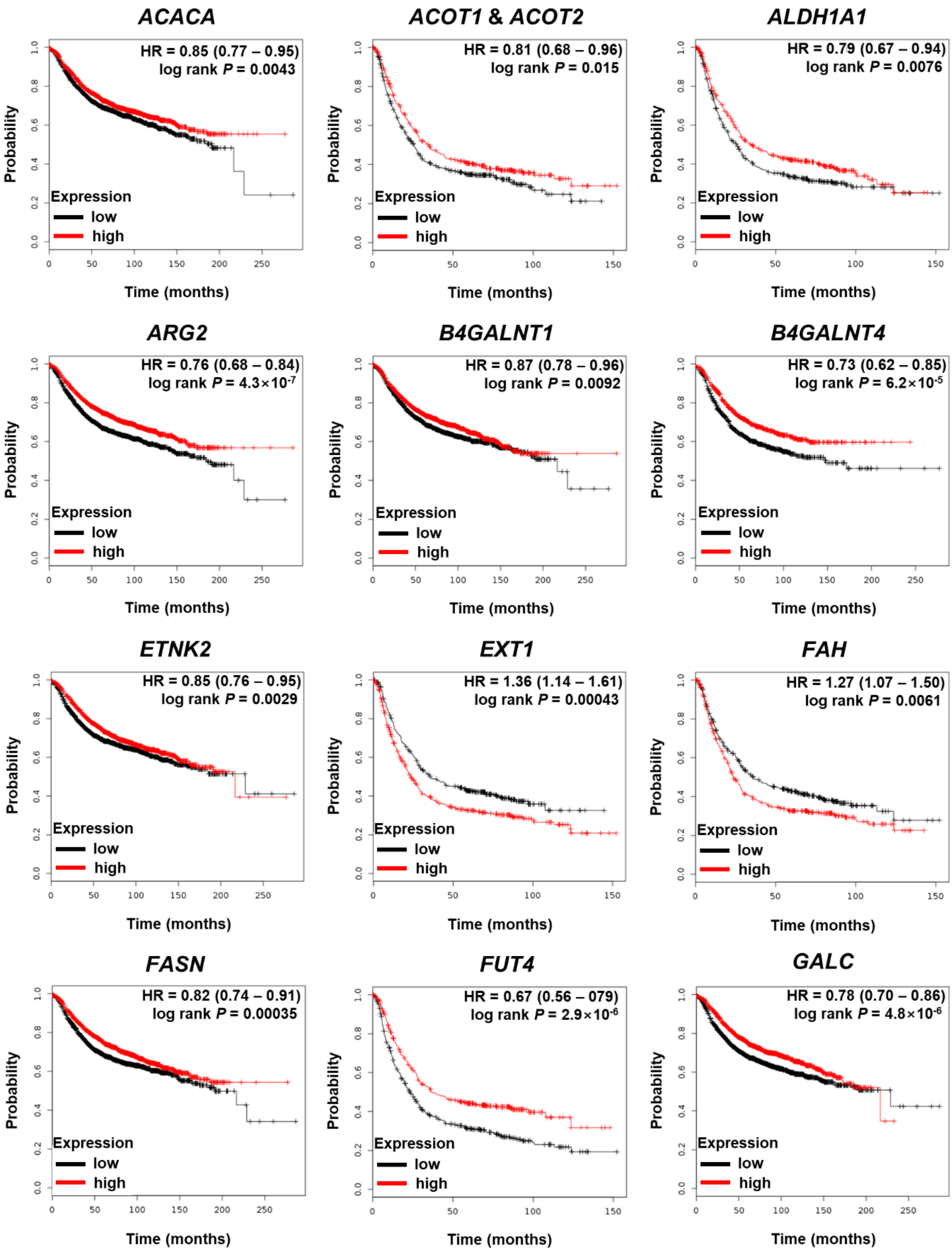
ACACA
HR = 0.85 (0.77 – 0.95)
log rank P = 0.0043
ACOT1 & ACOT2
HR = 0.81 (0.68 – 0.96)
log rank P = 0.015
ALDH1A1
HR = 0.79 (0.67 – 0.94)
log rank P = 0.0076
ARG2
HR = 0.76 (0.68 – 0.84)
log rank P = 4.3×10-7
B4GALNT1
HR = 0.87 (0.78 – 0.96)
log rank P = 0.0092
B4GALNT4
HR = 0.73 (0.62 – 0.85)
log rank P = 6.2×10-5
ETNK2
HR = 0.85 (0.76 – 0.95)
log rank P = 0.0029
EXT1
HR = 1.36 (1.14 – 1.61)
log rank P = 0.00043
FAH
HR = 1.27 (1.07 – 1.50)
log rank P = 0.0061
FASN
HR = 0.82 (0.74 – 0.91)
log rank P = 0.00035
FUT4
HR = 0.67 (0.56 – 079)
log rank P = 2.9×10-6
GALC
HR = 0.78 (0.70 – 0.86)
log rank P = 4.8×10-6
Expression
low
high
Probability
Time (months)

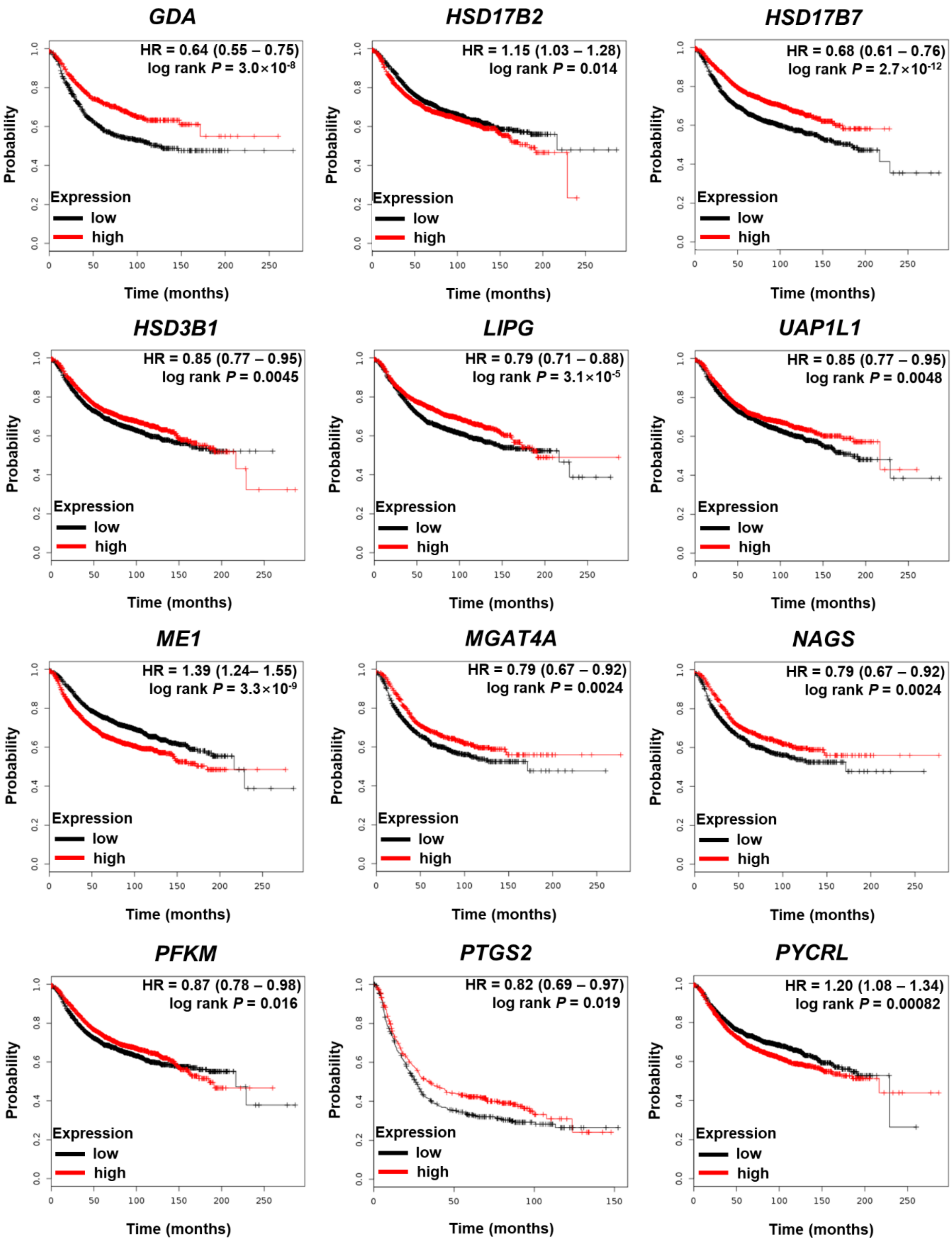
GDA
HR = 0.64 (0.55 – 0.75)
log rank P = 3.0×10-8
HSD17B2
HR = 1.15 (1.03 – 1.28)
log rank P = 0.014
HSD17B7
HR = 0.68 (0.61 – 0.76)
log rank P = 2.7×10-12
HSD3B1
HR = 0.85 (0.77 – 0.95)
log rank P = 0.0045
LIPG
HR = 0.79 (0.71 – 0.88)
log rank P = 3.1×10-5
UAP1L1
HR = 0.85 (0.77 – 0.95)
log rank P = 0.0048
ME1
HR = 1.39 (1.24– 1.55)
log rank P = 3.3×10-9
MGAT4A
HR = 0.79 (0.67 – 0.92)
log rank P = 0.0024
NAGS
HR = 0.79 (0.67 – 0.92)
log rank P = 0.0024
PFKM
HR = 0.87 (0.78 – 0.98)
log rank P = 0.016
PTGS2
HR = 0.82 (0.69 – 0.97)
log rank P = 0.019
PYCRL
HR = 1.20 (1.08 – 1.34)
log rank P = 0.00082
Expression
low
high
Probability
Time (months)

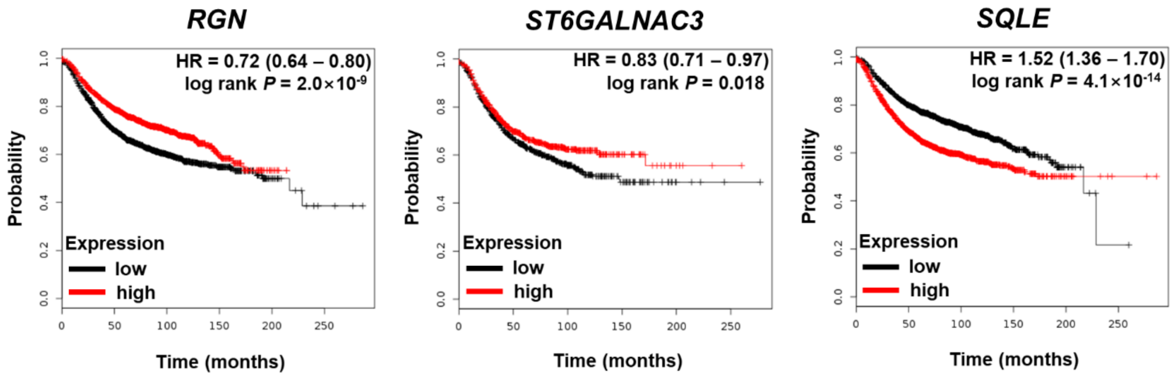

Figure S2. Kaplan─Meier survival plots of gastric cancer (GC) for metabolic genes significantly down-regulated in EBVaGC. HR: hazard ratio.

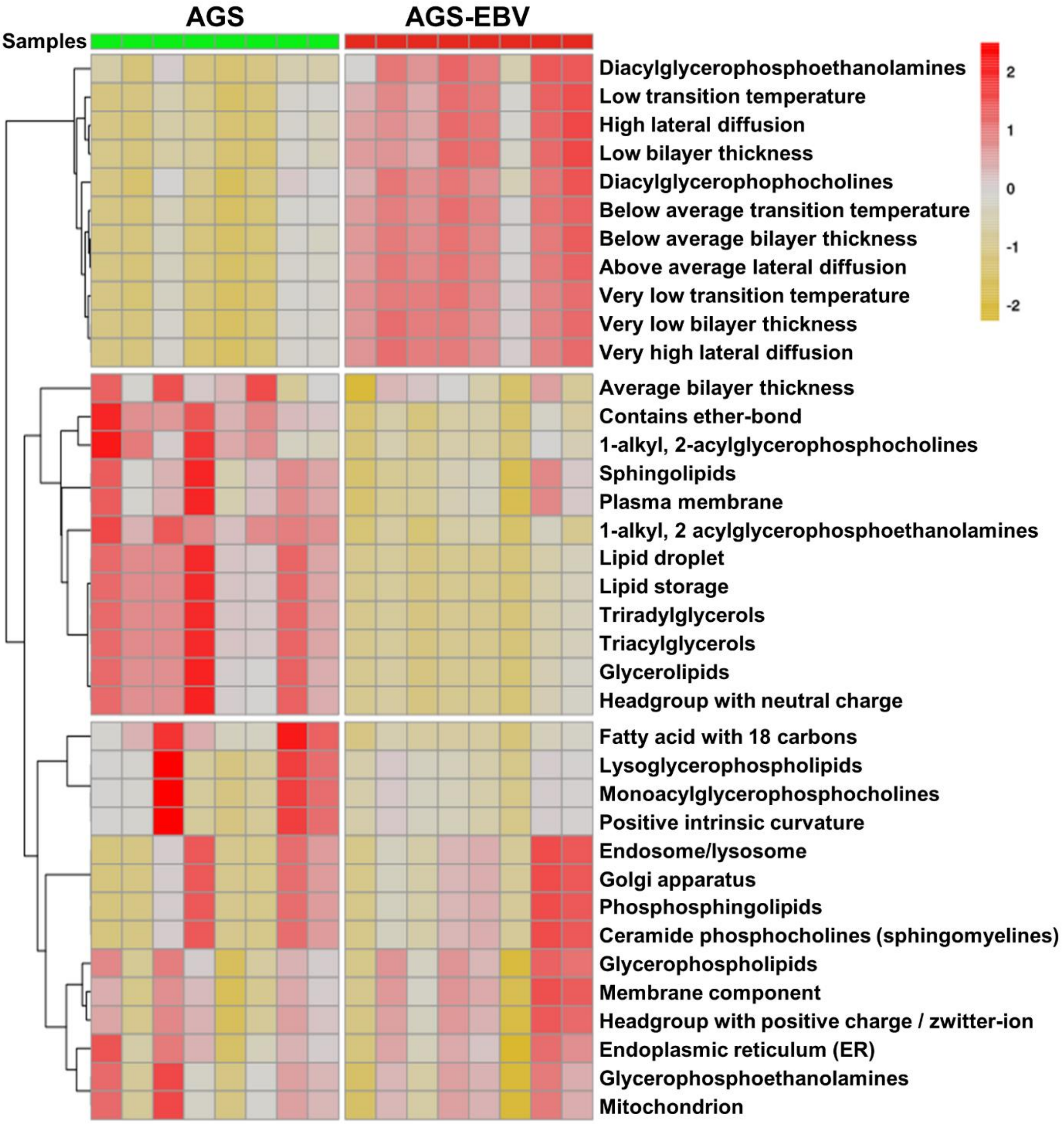

Figure S3. Heatmap of pathway enrichment analysis based on lipids significantly altered in EBVaGC.

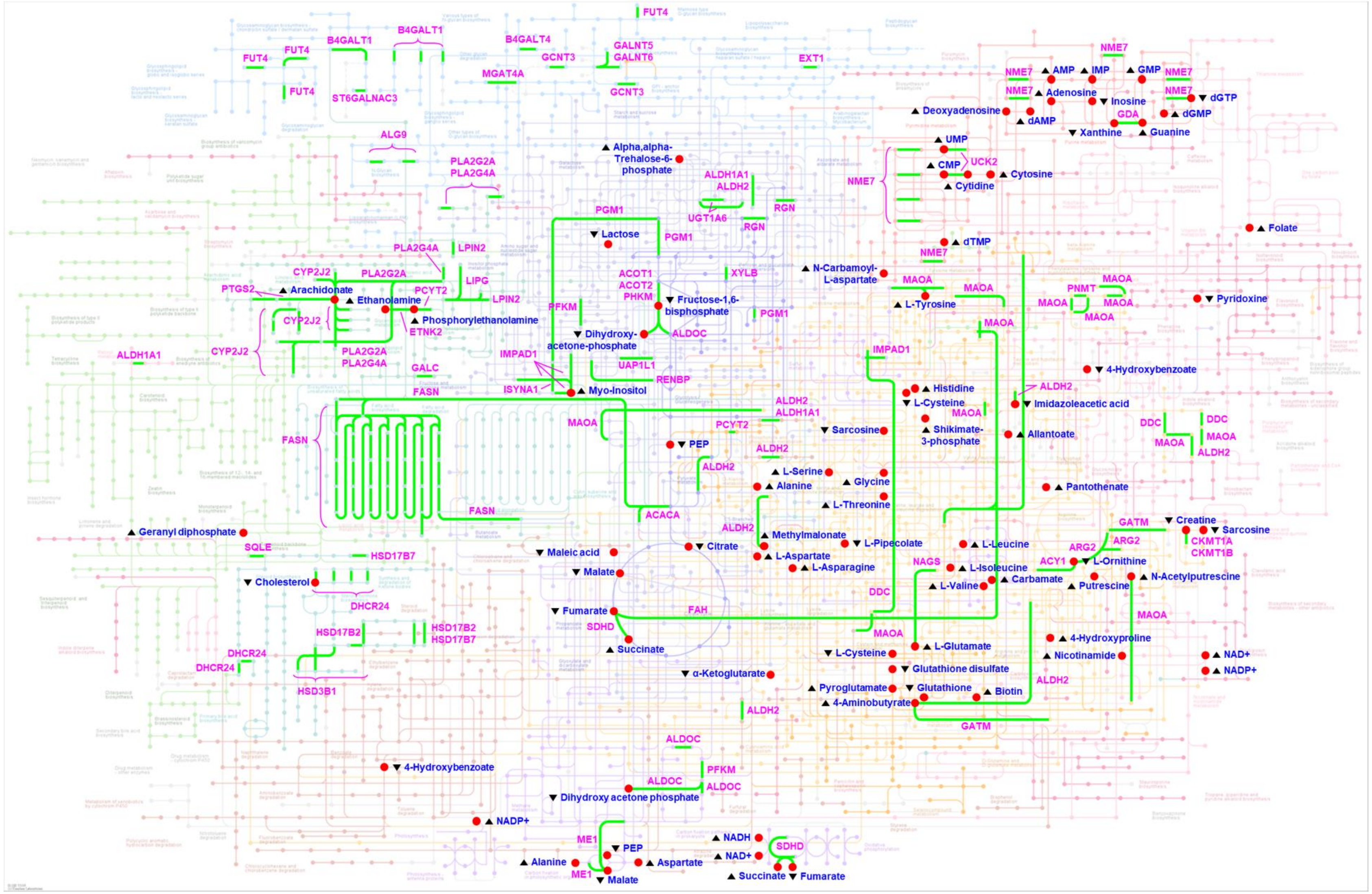

Figure S4. Metabolic pathways with transcripts significantly downregulated and metabolites altered by EBV infection in gastric cancer.
